# Differences in Gall Induction of Flower-like Galls on *Haloxylon* by Psyllids (Hemiptera: Aphalaridae), and the Emergence of Corresponding Parasitoids

**DOI:** 10.3390/insects12100861

**Published:** 2021-09-23

**Authors:** Qian Zhao, Ling-Ling Jiang, Jie Guo, Dong-Kang Zhang, Hong-Ying Hu

**Affiliations:** 1 College of Life Science and Technology, Xinjiang University, Urumqi 830046, China; ajazhq@163.com (Q.Z.); Jlling_0727@163.com (L.-L.J.); guojie1824658155@163.com (J.G.); zhangdongkang0602@163.com (D.-K.Z.); 2 Xinjiang Key Laboratory of Biological Resources and Genetic Engineering, Urumqi 830046, China

**Keywords:** *Caillardia*, *Psyllaephagus*, parasitization indexes, lifespan, biological control

## Abstract

**Simple Summary:**

*Haloxylon* spp. have been infested by various gall inducers, and natural enemies that impact pest populations must be understood to build integrated pest control strategies affecting pest dynamics. However, limited studies report on the gall inducers and parasitoids of flower-like galls on *Haloxylon*, as well as the parasitic efficacy of the parasitoids. This study aimed to determine the main gall inducers that induced flower-like galls on *Haloxylon ammodendron* and *H. persicum*, and the parasitoid complex associated with them. In total, five species of gall inducers and three species of parasitoids were obtained from three types of flower-like galls on *Haloxylon*. Further, the parasitization indexes, emergence, and lifespan of parasitoids were also discussed. The present study may serve as a basis for understanding the importance of biological investigations of parasitoids in the gall inducers living in flower-like galls, which is important for pest control and the conservation of the ecological environment in the *Haloxylon* forest.

**Abstract:**

Saxaul is a kind of dominant perennial psammophyte that widely distributes in arid and semi-arid desert areas, and it has multiple functions in preventing desertification, especially in windbreak and sand fixation. Various gall inducers induce galls on the saxaul, including the flower-like gall. Parasitoids have great potentiality in controlling gall inducers. However, studies about gall inducers and parasitoids of flower-like galls on *Haloxylon,* as well as the parasitic efficacy of the parasitoids, are rarely reported. In this study, the flower-like galls were observed on *Haloxylon ammodendron* and *H. persicum* in Fukang, Xinjiang, China. Two types of flower-like galls were found on *H. ammodendron*, while only one type was found on *H. persicum*. In total, five species of gall inducers and three species of parasitoids were obtained from the galls mentioned above. All the galls were induced by *Caillardia* (Hemiptera: Aphalaridae), which were mostly bivoltine in Fukang. Besides, their parasitoids *Psyllaephagus* *caillardiae* and *P. longiventris* could be observed on all the types of galls. Additionally, correlative studies on the parasitization indexes demonstrated that all the dominant parasitoids of diverse flower-like galls were *P. caillardiae*, which were slightly more in number than the *P. ogazae* discovered in the flower bud-like galls. In addition, the relevance between the emergence or lifespan of parasitoids and temperature was also investigated. The results showed that the number of parasitoids emerging decreased rapidly after a period of enhancement with the increase of temperature, including an optimum temperature, while the lifespan of wasps gradually shortened with the temperature rising. Our results highlight the importance of the biological investigation of parasitoids in the gall inducers lived in closed galls, which may provide critical evidence for us to understand its potential application in biological control.

## 1. Introduction

Saxaul (*Haloxylon* spp. Bunge) is one of the most widely distributed sub-shrublets or shrubs in arid and semi-arid desert areas worldwide, with properties of drought resistance, cold tolerance, and strong adaptability to suit windbreak plantations and sand fixations [1,2]. Globally, 11 *Haloxylon* species have been recorded, of which two are distributed in Xinjiang (Xinjiang Uyghur Autonomous Region of China): the cosmopolitan *Haloxylon ammodendron* (C. A. Mey.) Bunge and *H. persicum* Bunge, a native species, is only distributed in the Junggar Basin in northern Xinjiang [3]. Besides, saxaul plays important roles in adjusting the vegetation climate and increasing biodiversity [2]. Thus, it is of profound significance to protect and rejuvenate the *Haloxylon* plant to control desertification and maintain the ecological balance in the region.

**Figure 1:**
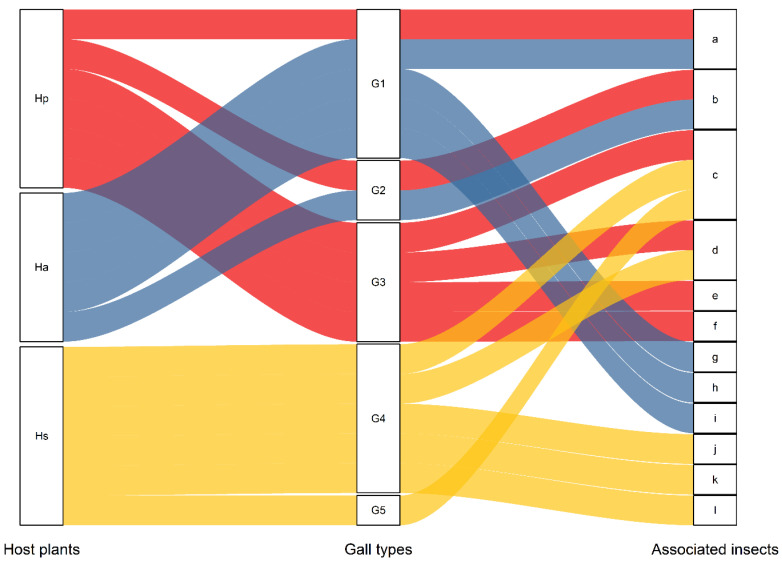
The relationships between the types of flower-like galls and their associated insects on three species of *Haloxylon* Worldwide. Hp: *H. persicun,* Ha: *H. ammodendron*,Hs: *H. salicornicum*; G1: leafy-bracted galls, G2–G4: flower-like galls, G5: spike-like galls; a-l indicate 12 species of associated insects. a: *C. notata*, b: *Aceria haloxylonis*, c: Pteromalidae, d: Thrips, e: Psyllidae, f: Pseudococcidae, g: *C. robusta*, h: *C. azurea*, i: *C. nana*, j: Pyralidae, k: Acari (Mites), l: Salticidae.

In addition to human activities, pests contribute significantly to the degradation of desert ecosystems. A variety of galls have been observed on the assimilated shoots, stems, and branches of seven species of *Haloxylon* spp. globally [4,5,6,7,8]. Five types of associated flower-like galls based on morphology have been identified on *Haloxylon* spp. (Figure 1) [4,9,10]. Leafy-bracted galls (G1) and flower like galls (G2) appeared with high frequency on *H. ammodendron*, while the leafy-bracted galls (G1) and flower like galls (G2 and G3) appeared with high frequency on *H. persicum* [8,9,10].

More than 17 species of gall inducers have been recorded that damage *Haloxylon* forest in the world [4,5,6,7,8,9,10]. These flower-like galls on *H. persicum* and *H. ammodendron* were associated with various pests that mainly belong to Psyllidae, Pyralidae, Pseudococcidae, and Thripidae. Moreover, psyllids are globally known as the major gall inducers on various organs of cultivated plants and forest trees, which are highly dependent on their host [11,12,13,14,15,16,17]. They have been recorded from the galls on *Haloxylon* frequently. At least four gall-forming species belonging to the genus *Caillardia* Bergevin (*C. robusta*, *C. azurea*, *C. nana*, and *C. notata*) were recorded and identified as the most harmful pests injuring *Haloxylon aphyllum* and *H. persicum* [18,19,20,21,22,23]. These species were first found in Kazakhstan and fed on the young twigs and leaves of *Haloxylon* [19,20]. These species showed a very distinct sexual dimorphism; they wintered in the egg phase and had two generations a year, but the gall type they induced was not reported. In China, these species were recorded from the leafy-bracted galls on *H. ammodendron* [9]. Consequently, these studies only illustrated that *Caillardia* spp. could make galls on *Haloxylon*, but little information is available about the species of the dominant gall inducer of each type of galls.

The control of gall inducers is important for the protection of the saxaul forest. However, the use of chemical insecticides, the dominant control method of gall inducers, has achieved limited success. It is also not a sustainable measure as it causes toxicities to beneficial non-target organisms [24,25]. Thus, the adoption of an integrated pest management (IPM) strategy is better alternative for sustainable pest control [26,27,28]. Biocontrol is a promising component of IPM which involves the use of parasitic natural enemies to control pests, and several studies have reported on the biocontrol potentials of parasitoids [29]. For instance, the host-specific parasitoid *Torymus sinensisis* (Hymenoptera: Torymidae) was reported as an effective biological control agent of the chestnut gall wasp *Dryocosmus kuriphilus* Yasumatsu (Hymenoptera: Cynipidae) [30,31,32,33]. The species composition of insects, degree of pest damage, and abundance of natural enemies have been noted as primary factors affecting IPM strategy [26,27,29,34]. The species *Psyllaephagus caillardiae* Sugonjaev and *P. ogazae* Sugonjaev were firstly reported as parasitoids of the scanty gall psyllids, *Caillardia robusta*, *C. azurea*, and *C. notata* which formed galls on *H. persicum* and *H. ammodendron* [35] Likewise, *Psyllaephagus* sp. (Encyrtidae) was reported as the parasitoid of *C. robusta*, *C. azurea*, *C. nana*, and *C. notata* which caused galls on *H. ammodendron* and *H. persicum* in the Gurbantunggut Desert in Xinjiang, China [9]. A new species of *Aprostocetus (Aprostocetus) essugonjaevi* (Hymenoptera, Eulophidae: Tetrastichinae) was recorded in galls induced by the psyllids *Caillardia* spp. on *H. persicum* and *H. ammodendron* in Uzbekistan [36]. However, the species resources of parasitoids of flower-like galls still lack systematic and comprehensive research in Northern Xinjiang.

Based on the information presented above, although our preliminary studies have found that more than one type of flower-like galls exist on *Haloxylon*, the research on indigenous gall inducers and their biological control is still in the preliminary stage. It is important to understand that the major pest species and their native natural enemy communities are essential for the further development of IPM program. Thus, the present study was conducted to evaluate the occurrence and infestation level of flower-like galls on *Haloxylon* and to determine the species and biological characteristics of the gall inducers and their parasitoids. The results may provide valuable information for the management and protection of the desert vegetation dominated by *Haloxylon*.

## 2. Materials and Methods

### 2.1. Study Sites and Sampling Collections

Two collection sites (Table 1) with saxaul trees naturally infested with galls were established to study the biological properties of the gall inducers and their parasitoids in Fukang city, Xinjiang, China, in 2018. Flower-like galls on *H. ammodendron* were surveyed in site 1 (S1), and on *H. persicum* in site 2 (S2). A five-point sampling method was used, as follows: three trees with heights of approximately 1–2 m were selected randomly from five sub-samples of trees from April to October. The number of fresh galls was counted. Then, 70–90 galls were randomly collected and taken to the laboratory for feeding and dissection.

### 2.2. Laboratory Rearing of the Galls

The field-collected galls were placed individually in glass vials (diameter 1.5 cm and length 10.0 cm) to monitor their development in the insect laboratory of the College of Life Sciences and Technology, Xinjiang University (ICXU). The galls were reared in controlled-climate chambers under four different temperatures (20, 26, 32, and 38 °C) at 65 ± 5% RH, with natural and fluorescent lighting of approximately 14:10 h (L:D), from April to October 2018.

The galls were checked every day and the emergence of gall inducers and the corresponding parasitoid adults were recorded and separated according to their taxa and sex. Sex ratio was further calculated as the mean percentage of female offspring for each type of parasitoid from the pest species that emerged in the laboratory. The emerged gall inducers and the corresponding parasitoids were reared in glass vials (diameter 1.5 cm and length 10.0 cm) with gauze. The gall inducers and parasitoids were fed with absorbent cotton soaked with water and 15% honey water, respectively. The lifespans of gall inducers and parasitoids were recorded every day until they all died. The dead gall inducers and parasitoids were stored in 100% ethanol for future identification.

### 2.3. Identification of Gall Inducers and Parasitoids

All specimens were maintained in 100% ethanol and subsequently air dried. Morphological terminology of gall inducers follows the taxonomic literature of Loginova and Parfentiev [19,20] and Li et al. [9], and morphological terminology of parasitoids follows the taxonomic identification keys by Noyes and Hayat [37] and Gibson et al. [38]. The parasitoids were first identified to the family level and then to the genus level. Then, the selected specimens of each morphospecies of parasitoids were identified by S. V. Triapitsyn, a professional taxonomist focusing on several families of Chalcidoidea at the Entomology Research Museum, University of California, Riverside, CA, USA (UCRC). All the specimens were deposited in ICXU.

Molecular analyses for the gall inducers and parasitoids were performed to confirm some morphological identification, and also to determine the correspondence between the gall inducers and parasitoids of the flower-like galls on *Haloxylon*. Genomic DNA was extracted following the method of Muhetaier et al. [39], and the barcode region of the *cytochrome oxidase I* (*COI*) gene was amplified using PCR primers for Psyllids C1–J1709 and HCO2198. The PCR primer FWPTF1 and Lep-R1 worked well for wasps [40]. The PCR primer 28sF3633 (5′-TACCGTGAGGGAAAGTTGAAA-3′) and 28sR4076 (5′-AGACTCCTTGGTCCGTGTTT-3′) were used to amplify the D2 domain of 28S (28SD2) nuclear ribosomal DNA (rDNA) [41]. The PCR was performed in a 25 μL reaction volume: 2 μL of DNA, 8.5 μL molecular grade water, 1 μL of each primer (0.3 μM each), 12.5 μL 2 × Taq Master Mix (Dye Plus) (Vazyme, Nanjing, China). The thermocycling conditions followed that of Triapitsyn et al. [42] and Morse et al. [43].

Amplification was confirmed by gel electrophoresis and PCR products were used for direct sequencing in both directions at Sangon Biotech Company, Shanghai, China. The sequences were compared to those in the GenBank database using the Basic Local Alignment Search Tool (http://www.ncbi.nlm.nih.gov/BLAST accessed on 15 June 2021). All sequences that showed a similarity score lower than 99% were lodged in the GenBank database. All residual DNA was archived in ICXU.

### 2.4. Parasitization Indexes and Eclosion Rates of Parasitoids in Flower-like Galls of Haloxylon

The number of gall inducers and parasitoids from the flower-like galls were recorded after they were dissected. The parasitization indexes and eclosion rate of the parasitoids were also determined. The parasitization indexes for each parasitoid species included the following [44]: (1) discovery efficiency (DE) which was calculated as (the number of flower-like galls with parasitoids/the number of collected flower-like galls) × 100, (2) exploitation efficiency (EE) which describes the ability of parasitoids to exploit the gall-inducers. It was calculated as (the number of parasitized gall-inducers in the flower-like galls/the total number of the gall inducers and parasitoid in the parasitized flower-like galls) × 100, (3) parasitoid impact (PI) which describes the efficacy of a parasitoid in reducing the gall inducers. It was calculated as (the number of parasitoids in flower-like galls/the total number of gall inducers and parasitoid in the flower-like galls) × 100, and (4) relative importance (RI) [45] was determined using the following formula: RI = [(the number of flower-like galls with parasitoids/the number of collected flower-like galls) × (the number of parasitoids in flower-like galls/total number of gall inducers and parasitoids in the flower-like galls)] × 100 (RI > 10 was considered “very frequent”; 9.99 ≥ RI > 1.0 was considered “frequent”; 1 ≥ RI ≥ 0.09 was considered “scarce or occasional species” and RI < 0.09 was considered “rare”). The eclosion rate was calculated as (the number of parasitoids which emerged from flower-like galls/the total number of gall inducers parasitized by the same parasitoids in the same flower-like galls) × 100.

### 2.5. Statistical Analysis

All analyses were performed using Prism 8.0 (Graphpad Software, San Diego, CA, USA) and assessed for normality (Shapiro–Wilk test) and variance, when appropriate. Sample sizes were chosen according to standard practice in the field. A two-tailed Student’s *t* test was used to determine statistical significance between two groups. For multiple comparisons, one-way ANOVA followed by Tukey’s post-test was used. The significance level was set at *p* < 0.05. Data are indicated as mean ± S.E.M of at least three independent experiments.

## 3. Results

### 3.1. The Occurrence of Flower-like Galls of Haloxylon

**Figure 2:**
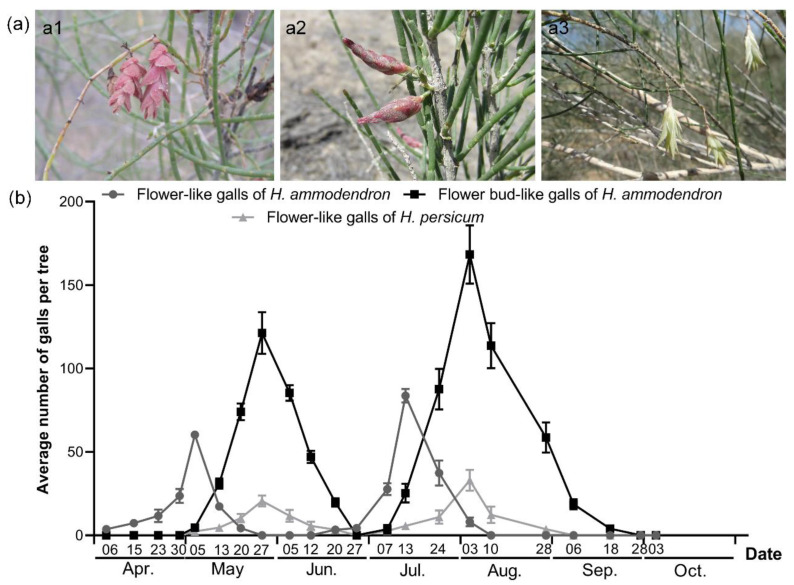
The types (**a**) and occurrence (**b**) of flower-like galls on *Haloxylon* in Fukang, Xinjiang. (**a1**) The flower-like gall on *H. ammodendron*; (**a2**) The flower bud-like gall on *H. ammodendron*; (**a3**) The flower-like gall on *H. persicum*.

Three types of flower-like galls were recorded on *Haloxylon* through the field surveys. The first type was recorded on *H. ammodendron* and was characterized by bracts that unfolded like a flower. The second type was a flower bud-like gall which was also recorded on *H. ammodendron*. It was characterized by pink and yellow-green bracts that did not unfold. The third type was a flower-like gall recorded on *H. persicum* (Figure 2a). It was smaller than the flower-like gall recorded on *H. ammodendron* and was characterized by pale green bracts with slightly pink tips that spread like flowers. The galls occurred twice a year; the first period was from late April to late June, and the second period was from early July to mid-September. The galls dried up after aging, appearing dark brown or yellowish-brown. The occurrence of all the flower-like galls in the succeeding generation was higher than that in the initial generation. The maximum number of the second generation of flower-like galls on *H. ammodendron* was recorded in mid-July (averaging 84 galls per tree), and that of flower bud-like galls on *H. ammodendron* and flower-like galls on *H. persicum* were recorded in early August (averaging 168 galls and 33 galls per tree, respectively) (Figure 2b).

### 3.2. Species of Gall Inducers and Parasitoids of Flower-like Galls on Haloxylon

A total of 843 (the number of flower-like galls and flower bud-like galls of *H. ammodendron*) and 286 (the number of flower-like galls of *H. persicum*) of flower-like galls on *Haloxylon* were collected, respectively, at the two sites (S1 and S2) surveyed in 2018 in Fukang. Five species of gall inducers of the genus *Caillardia* (Hemiptera: Aphalaridae), were recorded from the flower-like galls on *Haloxylon*. *Caillardia anabasidis* and *C. azurea* were recorded from the flower-like galls on *H. ammodendron*, *C. robusta*, and *C. nana* were recorded from the flower bud-like galls on *H. ammodendron*, and only *C. notata* was recorded from the flower-like galls of *H. persicum* (Table 2). All psyllid species were mostly bivoltine, and they overwintered at the adult stage in the dead grass or the bark of the *Haloxylon*.

Three species of parasitoids of the genus *Psyllaephagus* (Hymenoptera: Encyrtidae), were recorded from the flower-like galls on *Haloxylon* (Table 2). All were solitary koinobiont primary nymphal endoparasitoids of *Caillardia* spp. *P. caillardiae*, *P. longiventris*, and *P. ogazae* were recorded from both flower-like galls and flower bud-like galls on *H. ammodendron*. The number of *P. ogazae* recorded from the flower bud-like galls of *H. ammodendron* (♀53♂54) was higher than that recorded from the flower-like galls on *H. ammodendron* (♀1♂0). However, only *P. caillardiae* and *P. longiventris* were recorded from the flower-like galls on *H. persicum*. Their numbers were lower than in the galls on *H. ammodendron*. In addition, the number of female wasps was slightly higher than that of the male wasps of each species of parasitoids emerging from the corresponding galls.

### 3.3. Parasitization Indexes of Parasitoids Recorded from Flower-like Galls on Haloxylon

The different parameters of parasitization (discovery efficiency, exploitation efficiency, parasitoid impact, and relative importance) are given in Table 3. There were differences among all the parasitization indexes of the parasitoids. Overall, all the parasitization indexes of *P. caillardiae* were significantly higher than that of *P. ogazae* (F = 4.019, *p* = 0.0280) and *P. longiventris* (*p* = 0.0072). The parasitoid impact and relative importance of *P. caillardiae* were 32.74% and 10.04, respectively. The relative importance of *P. caillardiae* peaked at 44.44% (in flower bud-like galls of *H. ammodendron* collected on 24 July), that of *P. ogazae* was 21.49% (in flower bud-like galls of *H. ammodendron* collected on 28 August) and that of *P. longiventris* was 11.11% (in flower bud-like galls of *H. ammodendron* collected on 24 July). The results indicated that *P. caillardiae* was the dominant parasitoid of flower-like galls on *Haloxylon*.

### 3.4. Effect of Temperature on the Emergence of Parasitoids from Flower-like Galls on Haloxylon

**Figure 3:**
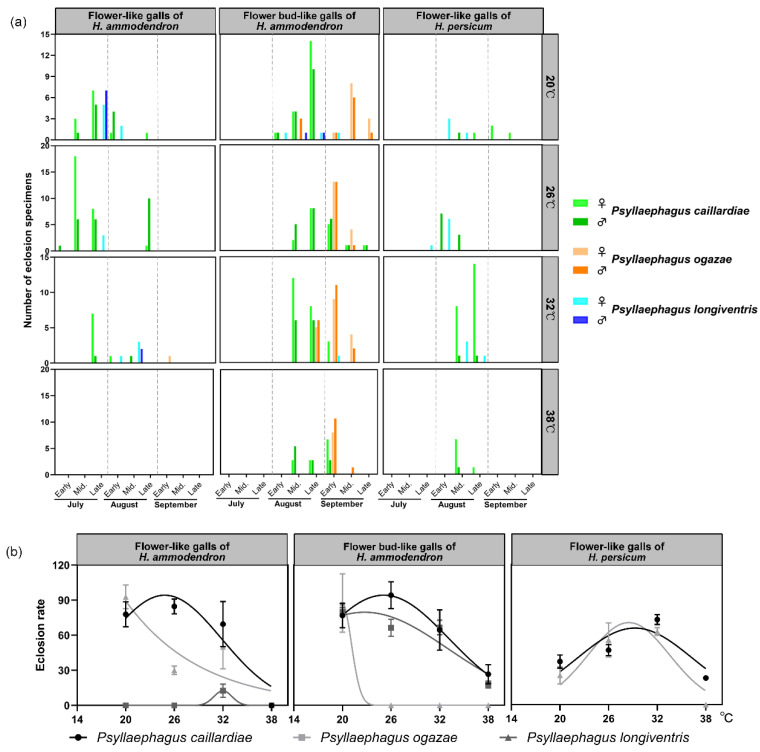
Emergence periods (**a**) and emergence rate effects (**b**) of parasitoids of flower-like galls on *Haloxylon* at four different temperatures of 20, 26, 32, and 38 °C. The emergence of individuals from flower-like galls was investigated daily under lab conditions in Fukang, China. Midpoints mean the average value. The regression line shows the trend of emergence rate with temperature.

**Figure 4:**
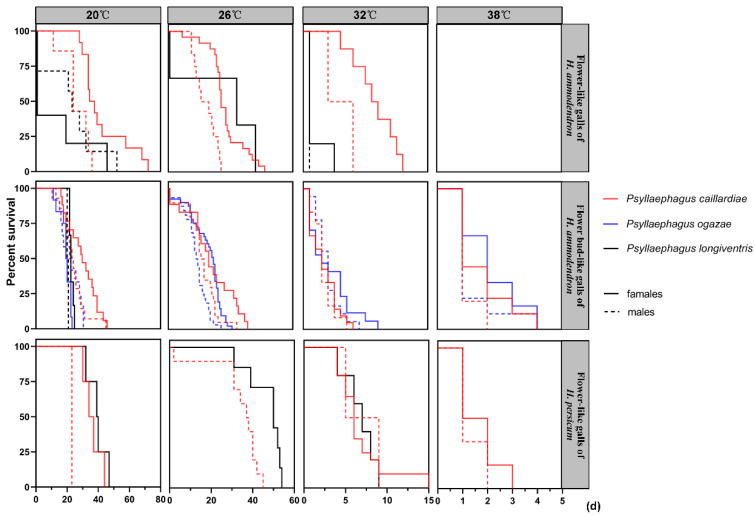
Survival curves of parasitoids of flower-like galls on *Haloxylon* at four different temperatures of 20, 26, 32, and 38 °C.

The number of emerged parasitoids from the flower bud-like galls was the highest (♀125♂112) among the three types of flower-like galls. The number of emerged parasitoids from the flower-like galls peaked at 26 °C (♀71♂68) and lowered at 38 °C (♀21♂18) (Figure 3a). The parasitoids recovered from flower-like galls of *H. ammodendron* emerged earlier than that from other flower-like galls; *P. caillardiae* emerged earlier than *P. ogazae*, which rarely emerged from other flower-like galls, but from the flower bud-like galls of *H. ammodendron* (Figure 3a,b and Figure 4). The emergence rate of parasitoids recovered from flower-like galls of *Haloxylon* showed a rising tendency at first and then decreased with an increase in temperature from 20 °C to 38 °C. The highest eclosion rate was recorded at 26 °C, and the minimum at 38 °C (Figure 3b). The eclosion rate of parasitoids recovered from flower bud-like galls of *H. ammodendron* was the highest compared to that of parasitoids from the other two types of flower-like galls. The optimum temperature for eclosion was between 26 °C and 32 °C.

### 3.5. Effect of Temperature on the Lifespans of Adult Parasitoids Recovered from the Flower-like Galls on Haloxylon

The lifespans of the three species of *Psyllaephagus* showed differences, and they were affected by temperature and the type of flower-like galls (Figure 4). The lifespans of adult parasitoids that emerged from flower-like galls on *Haloxylon* showed a shortened trend with an increase in temperature. The lowest number and the shortest lifespan (less than five days) of parasitoids were recorded at 38 °C. The average lifespans expectancy of *P. caillardiae* and *P. ogazae* at 20 °C were 36.64 days and 25.79 days, respectively. They were, respectively, 1.46 days and 1.81 days at 38 °C. The average lifespan of *P. longiventris* at 20 °C was 28.94 days. When the temperature rose to 38 °C, no living *P. longiventris* was observed.

Moreover, the females of the three species of *Psyllaephagus* recorded from flower-like galls on *Haloxylon* had longer lifespans than males at 20 °C, except for *P. longiventris* that emerged from the flower-like galls on *H. ammodendron* and *P. ogazae* that emerged from the flower bud-like galls on *H. ammodendron*. The lifespan of female *P. caillardiae* recorded from the flower-like galls on *H. ammodendron*, was longer than that of female *P. longiventris* at all the given temperatures. The lifespan of female *P. longiventris,* recorded from the flower bud-like galls on *H. ammodendron* at 20 °C, was significantly longer than that of males (χ^2^ = 11.20, *p* = 0.0389). However, there were no significant differences in lifespans between female and male *P. ogazae* or *P. caillardiae* which were recorded from the flower-like galls on *H. ammodendron* at all the temperatures. Only female *P. longiventris* emerged from the flower-like galls on *H. persicum*. However, there were no significant differences in lifespans between females and males of each parasitoid species from flower-like galls on *H. persicum* at all temperatures (Figure 4).

### 3.6. Gall Inducers and Parasitoid Species Characterization

Molecular analyses confirmed the identity of gall inducers and parasitoids that emerged from the flower-like galls on *Haloxylon*. Since sequences of five species of *Caillardia* and three species of *Psyllaephagus* were not present in the GenBank database, all sequences obtained from specimens identified by the morphological analysis were deposited into the GenBank database (Table 4). A total of 33 sequences were obtained. These included the *CO1* and 28S sequences of *Caillardia*, except that of *CO1* of *C. nana*. For *Psyllaephagus caillardiae* only the *CO1* sequence was obtained, but none from the other two wasps. 

## 4. Discussion

In this study, we found that the galls on the *Haloxylon* spp. could be divided into three types. The galls of *H. ammodendron* especially could be divided into flower-like galls and flower-bud like galls based on whether their bracts expanded or not. The present study indicated that the three types of flower-like galls on the *Haloxylon* spp. were associated with different gall inducers, all of which belonged to the genus *Caillardia*. Moreover, the gall inducers of the flower-like galls and flower bud-like galls, which were two similar kinds of flower-like galls on the same saxaul plant species, were also different. However, a previous study reported that flower-like galls and flower bud-like galls on *H. ammodendron* were grouped into the same category, and four psyllid species (*C. robusta, C. azurea, C. nana,* and *C. notata*) were the inducers of these galls on *H. ammodendron* [9]. In addition, our study demonstrated that *C. notata* was the only inducer of flower-like galls on *H. persicum*. This is consistent with the result of the previous study [9].

Psyllid is a kind of sucking insect highly dependent on its host [11,12,13,14,15,16,17]. Many gall inducers in Psylloidea are highly host-specific and occur in all of the currently recognized families [46,47,48,49]. In present study, only species of *Caillardia* induced galls with multiple flower-like scales on *H. ammodendron* and *H. persicum*, while they were reported as gall inducers on *Haloxylon* spp. previously [18,19,20,21,22,23]. Interestingly, two psyllids species shared the same type of galls on *H. ammodendron* sympatrically, although different psyllid species coexisting on the same host plant usually used at least slightly different ecological niches [50]. Based on the numerical superiority and degree of damage of *C. anabasidis* and *C. nana*, we suggested that they were dominant gall inducers of the flower-like galls and flower bud-like galls, respectively.

Due to the very distinct sexual dimorphism of *Caillardia* spp. and slight morphological differences among three species of *Psyllaephagus* spp., it was difficult to identify the species only by morphological characters. As such, determining their species identities using genetic analyses was identified as one of the priorities within a study with broader sampling [51,52]. As a routine procedure, all species reared from different galls were accurately identified by the combination of both molecular and morphological characters. The consequence suggested that DNA barcoding of *CO1* and 28S specific fragments was feasible for the rapid identification of *Caillardia* and *Psyllaephagus*. However, the corresponding relationships between gall inducers and their parasitoids remain to be determined. 

Generally, parasitization indexes were widely used to evaluate the parasitic efficacy of egg parasitoids [45,53]. In this study, we applied the indexes to evaluate the overall efficiency of nymphal parasitoids from flower-like galls for the first time. Among them, the exploitation efficiency (EE) was higher than the discovery efficiency (DE). In most cases, the EE was 100% since only one parasitized psyllid was reared from one gall. Flower-like galls of *H. ammodendron* collected on 13 July and flower bud-like galls collected on 3 August and 10 August had lower EE. A reasonable explanation was that both gall inducers and galls occurred in abundance at these times normally. While some of these galls only contained a non-parasitized gall-inducing psyllid, others contained both parasitoid(s) and psyllid(s). The index, RI, indicated the frequency of parasitoids recorded from the galls, and it was used to determine the dominant parasitoids species from different galls. Our results demonstrated that *P. caillardiae* was the dominant parasitoid of flower-like galls on *Haloxylon*. However, dissection of the flower-like galls showed that it was difficult for psyllids to develop from young nymphs into adults in laboratory conditions, and also difficult to determine from their external appearances whether young nymphs had been parasitized. This could result in the underestimation of the actual parasitic rate in the wild. Intriguingly, the parasitoid impact (PI) of *P. ogaza* was the lowest in mid-to-late July, while that of *P. caillardiae* came to the highest during the same periods, indicating that there may be parasitic competition between them.

The results showed that the parasitic period and the eclosion time of the parasitoids were consistent with the occurrence of corresponding galls and the developmental duration of gall inducers and their parasitoids in the three types of flower-like galls. Based on the occurrence period of these galls, the parasitic period of *P. caillardiae* was earlier than that of *P. longiventris*, and *P. ogazae* parasitized last. The emerging period of parasitoids (especially the dominant parasitoid *P. caillardiae*) coincided with the high incidence of the corresponding galls on *Haloxylon*. In detail, the period of parasitoids emerged from the flower-like galls of *H. ammodendron* mainly ranged from mid-July to early August, which coincided with the high incidence of the galls in mid-July. However, the parasitic rate of parasitoids was the lowest at the early stage of gall formation than that at other stages. It indicated that the parasitoids attacked the gall inducers most frequently after the early stage of galls formation following the maturation of galls and when the nymph of *Caillardia* had developed.

The galls provide shelters for the gall inducers and increase the difficulty for the parasitic natural enemies to parasitize in the hosts [54]. Anecdotal evidence suggests that the parasitic rates of wasps decrease with increased gall size, while the eclosion rate of adults increases [55,56,57]. However, in this study, the parasitic rate (PI) of wasps on *H. ammodendron* was higher than that on *H. persicum*, and the eclosion rate showed the opposite, while the size of flower-like galls on *H. ammodendron* was bigger than those on *H. persicum*. The epidermal cells of integument of flower-like galls on *H. ammodendron* severely damaged by *Caillardia* spp. resulted in a loose arrangement and the exposure of vascular tissues [58]. Therefore, the flower-like galls were easier to pierce, compared to the severely lignified galls. Meanwhile, our previous study found that the ovipositor length of *Psyllaephagus* was about 0.39–0.82 mm, making it easier to pierce through the bracts of the galls (0.49 ± 0.22 mm) [59]. Nonetheless, it was unclear whether successful parasitism of parasitoid was related to the ovipositor length of parasitoid and the size of flower-like gall on *Haloxylon* and therefore deserved to be further studied.

Temperature is an important environmental factor that affects the life activities of insects [60,61]. The present study demonstrated that the eclosion rate and lifespans of parasitoids changed with an increase in temperature. When the temperature ranged from 20 °C to 38 °C, the eclosion rate of parasitoids increased first and then decreased, while their lifespans decreased gradually. This was consistent with the report about the composition of *Torymus* parasitoids found in the galls of chestnut trees and other related studies on parasitoids [62,63,64]. However, the biological characteristics (eclosion rate, the sex ratio and longevity) of parasitoids were influenced by many other internal factors (female wasp nutrition, egg nutrition, and ovipositing age of female wasps) and external factors (temperature, humidity, and light cycle) [33,65]. Therefore, the influences of these factors on the biological characteristics of the parasitoids recorded in this study need to be further evaluated.

Our findings highlight the importance of knowing the biological characteristics of the parasitoids of gall inducers. The results may contribute to an understanding of the need for their conservation and potential application in biological control. However, the parasitoids on *Haloxylon* were investigated only in native areas where the control efficiency of these gall inducers in Xinjiang was low (the highest parasitic rate was about 36%). Thus, an effective integrated pest management (IPM) program against the gall inducers remains to be further developed. It is recommended that a more comprehensive survey be conducted to identify more parasitoid species, and to assess the possibility of using these parasitoids to control pests. In addition, the mechanism underlying parasitoids search for their corresponding hosts as well as the nutritional relationships among galls, gall inducers, and parasitoids remain to be further investigated.

## Figures and Tables

**Table 1:** Survey sites of *Haloxylon* in Fukang in 2018.

Study Site	Coordinates	Altitude (m)	Habitat Type	Sampling Areas (m^3^)	Main Vegetation	Gall Collected
S1: Beishawo Desert of Fukang city in Xinjiang	E 87°53′6″ N 44°22′26″	374	Saline-alkali desertification	100	*Haloxylon* *Halimodendron* *Alhagi* *Salsola* *Tamarix*	Flower-like galls of *H. ammodendron*, Flower bud-like galls of *H. ammodendron*
S2: Fukang National Field Scientific Observation and Research Station for Desert Ecosystems in Xinjiang	E 87°55′45″ N 44°22′0″	408	Saline-alkali desertification	100	*Haloxylon* *Halimodendron* *Salsola* *Tamarix*	Flower-like galls of *H. persicum*

**Table 2:** Parasitoids and their hosts of flower-like galls on *Haloxylon* in 2018.

Type of Galls	Gall Inducers (Number of Adults)	Parasitoids (Number of Adults and Sex Ratio)	Generation of Gall Inducers	Overwintering Mode of Gall Inducers	Host Stage Attacked (Instar)	Habit of Parasitoids	Deworming State (Host)	Deworming State (Parasitoid)	Identifier
Flower-like galls of *H. ammodendron*	*C.anabasidis* (146) *C. azurea* (58)	*P. caillardiae* (♀47♂35) *P. ogazae* (♀1♂0) *P. longiventris* (♀14♂9)	2	Adults in the dead grass or the bark of the *H. ammodendron*	Nymph (III, IV)	Primary, solitary, endoparasitoid	Nymph	Adult	S.V. Triapitsyn and H.-Y. Hu
Flower bud-like galls of *H. ammodendron*	*C. robusta* (12) *C. nana* (189)	*P. caillardiae* (♀68♂56) *P. ogazae* (♀53♂54) *P. longiventris* (♀4♂2)	2	Adults in the dead grass or the bark of the *H. ammodendron*	Nymph (III, IV)	Primary, solitary, endoparasitoid	Nymph	Adult	S.V. Triapitsyn and H.-Y. Hu
Flower-like galls of *H. persicum*	*C. notata* (161)	*P. caillardiae* (♀32♂14) *P. longiventris* (♀15♂0)	2	Adults in the dead grass or the bark of the *H. persicum*	Nymph (IV)	Primary, solitary, endoparasitoid	Nymph	Adult	S.V. Triapitsyn and H.-Y. Hu

**Table 3:** Parasitization indexes and relative importance of the parasitoid species of flower-like galls on *Haloxylon*.

Temperature	Date	*Psyllaephagus caillardiae*				*Psyllaephagus ogazae*				*Psyllaephagus longiventris*			
		DE	EE	PI	RI	DE	EE	PI	RI	DE	EE	PI	RI
Flower-like galls of *H. ammodendron*	07/July/2018	23.38	96.67	18.81	4.61(F)	/	/	/	/	6.25	50.00	1.76	0.60(S)
	13/July/2018	54.29	74.69	40.87	22.89(VF)	0.72	25.00	0.48	0.01(S)	12.14	82.64	10.03	1.34(F)
	24/July/2018	35.19	95.83	34.33	12.08(VF)	7.41	100	5.97	0.44(S)	11.11	100.00	8.96	1.00(S)
	03/August/2018	16.67	100.00	16.67	2.78(F)	16.67	100.00	16.67	2.78(F)	/	/	/	/
	Total	32.38	91.80	27.67	10.59(VF)	8.27	75.00	7.71	1.08(F)	9.83	77.55	6.92	0.98(S)
Flower bud-like galls of *H. ammodendron*	24/July/2018	66.67	100.00	66.67	44.44(VF)	/	/	/	/	33.33	100.00	33.33	11.11(VF)
	03/August/2018	1.67	16.67	7.14	0.48(S)	1.67	16.67	3.13	0.21(S)	4.17	25.00	7.81	1.30(F)
	10/August/2018	32.58	75.76	26.38	9.21(F)	28.57	57.61	20.08	7.3(F)	/	/	/	/
	18/August/2018	58.97	100.00	59.80	36.54(VF)	16.67	75.00	16.49	3.80 F)	1.28	25.00	1.28	0.07(R)
	28/August/2018	23.89	100.00	24.16	6.37(F)	44.97	100.00	44.97	21.49(VF)	0.89	25.00	0.89	0.03(R)
	Total	36.76	78.49	36.83	19.41(VF)	22.97	62.32	21.17	8.21(F)	9.92	43.75	10.83	3.13 (F)
Flower-like gall of *H. persicum*	24/July/2018	20.00	100.00	20.00	0.04(R)	/	/	/	/	11.67	100.00	11.67	0.01(R)
	03/August/2018	25.73	100.00	25.73	0.07(R)	/	/	/	/	10.54	100.00	10.54	0.01(R)
	11/August/2018	54.63	98.53	55.47	0.30(S)	/	/	/	/	7.41	50.00	6.67	0.01(R)
	Total	33.45	99.51	33.73	0.14(S)	/	/	/	/	9.87	83.33	9.63	0.01(R)

Discovery efficiency = DE; Exploitation efficiency = EE; Parasitoid impact = PI; Relative importance = RI. RI ≥ 10, very frequent (VF); 9.99 ≥ RI > 1.0, frequent (F); 1 ≥ RI ≥ 0.09, scarce or occasional species (S); RI < 0.09, rare (R); /, absent.

**Table 4:** Sampling information and GenBank accession number for the deposited sequences generated from this study.

Species	No. Identified by Molecular Methods	Sex	Associated Host	*CO1* GenBank Accession No.	28S GenBank Accession No.
*P. caillardiae*	3	1♂2♀	Flower-like galls *H. ammodendron* Flower bud-like galls of *H. ammodendron*	MZ436006 MZ438311	MZ436078 MZ469911 MZ469912
*P. ogazae*	4	2♂2♀	Flower bud-like galls of *H. ammodendron*	/	MZ436075 MZ469916 MZ469909 MZ469905
*P. longiventris*	3	1♂2♀	Flower-like galls of *H. persicum*	/	MZ436079 MZ469917
*C. anabasidis*	3	2♂1♀	Flower-like galls *H. ammodendron*	MZ436072 MZ437083	MZ436073 MZ469908 MZ469903
*C. azurea*	3	2♂1♀	Flower-like galls *H. ammodendron*	MZ436003 MZ437085 MZ438310	MZ436076 MZ469907
*C. robusta*	4	1♂3♀	Flower bud-like galls of *H. ammodendron*	MZ436005	MZ436077 MZ469915 MZ469904
*C. nana*	3	2♂1♀	Flower bud-like galls of *H. ammodendron*	/	MZ436074 MZ469914 MZ469910
*C. notate*	5	3♂2♀	Flower-like galls of *H. persicum*	MZ436004	MZ436080 MZ469906 MZ469913
